# Detección de anticuerpos IgG frente a SARS COV2 en el suero y la leche materna de mujeres vacunadas

**DOI:** 10.1515/almed-2021-0083

**Published:** 2021-12-07

**Authors:** Sidra Sadiq, Faheem Arslan

**Affiliations:** Departamento de Química Clínica y Endocrinología, Rehman Medical Institute, Peshawar, Khyber Pakhtunkhwa, Pakistan; Departamento de Radiología, Pakistan Kidney and Liver Institute, Lahore, Punjab, Pakistan

**Keywords:** anticuerpos, COVID 19, SARS-CoV-2, vacunación materna

## Abstract

**Objetivos:**

A medida que la COVID 19 sigue extendiéndose, la transmisión de anticuerpos maternos frente al SARS COV2 durante la lactancia es una importante fuente de inmunización en los lactantes. Este fenómeno requiere estudios en profundidad, con el fin de mejorar las opciones de vacunación de estos candidatos. El propósito del presente estudio es evaluar la presencia de anticuerpos IgG frente a la proteína *Spike* del SARS-CoV-2 en la leche materna y el suero de madres lactantes tras su vacunación, así como establecer una correlación entre los niveles de anticuerpos en la leche y el suero materno.

**Métodos:**

En este estudio de cohortes realizado en el Rehman Medical Institute, en Peshawar (Pakistán), se incluyeron 180 mujeres en periodo de lactancia. Describimos la inmunogenicidad de 21 pacientes 21 días después de haber recibido la dosis de refuerzo de la vacuna. Se tomaron muestras de leche y suero materno para analizar la presencia de anticuerpos IgG frente a la proteína *Spike* del SARS-CoV-2 mediante inmunoensayo de electroquimioluminiscencia (ECLIA) (Elecsys Anti-SARS-CoV-2 S Roche, Suiza).

**Resultados:**

Se detectó claramente la presencia de anticuerpos IgG frente a la proteína *Spike* del SARS-CoV-2 en la leche materna de todas las participantes, y en el suero del 85% de las mujeres de la muestra (>0,8 IU/mL). Nuestro estudio revela que las madres lactantes pueden desarrollar fuertes reacciones inmunológicas frente al SARS-CoV-2 tras la vacunación.

**Conclusiones:**

Los niveles de anticuerpos frente al SARS-CoV-2 aumentaron significativamente en todas las participantes tras la vacunación. Así, los niveles de anticuerpos aumentaron una escala con respecto al periodo previo a la vacunación. Estos hallazgos muestran una correlación entre los niveles de anticuerpos frente a SARS COV2 en la leche y el suero materno. El monitoreo continuo de los títulos de anticuerpos demuestra que, tras la vacunación, se desarrolla una inmunidad humoral significativa frente a la infección por SARS-CoV-2.

## Introducción

Las mujeres en periodo de lactancia son un grupo de riesgo en el contexto de la vacunación contra la COVID -19, ya que pueden transmitir el virus a los neonatos y estos pueden experimentar los efectos secundarios de la vacuna. Sin embargo, no existen datos sobre la vacunación de este grupo de población. Sino pharm y Sinovac utilizan el virus COVID-19 desactivado para provocar una respuesta inmune empleando hidróxido de aluminio como adyuvante. Sino pharm y Sinovac han demostrado tener una efectividad del 73% (40.000 participantes) y el 83,5% (10.000 participantes), respectivamente, en ensayos de fase III [1], [2], [3]. El 7 de mayo de 2021 y el 1 de junio de 2021, respectivamente, la Organización Mundial de la Salud (OMS) aprobó el uso de las vacunas de Sino pharm y Sinovac en mujeres embarazadas [4, 5]. El gobierno chino también ha señalado la importancia de vacunar a este grupo de población [3, 6, 7]. Los estudios revelan la presencia de anticuerpos frente a SARSCoV-2 en la leche de madres infectadas [8], aunque existe escasa evidencia de la presencia de anticuerpos IgG frente a la proteína *Spike* del SARS-CoV-2 en la leche materna de mujeres vacunadas [9], [10], [11], [12], [13], [14].

Evaluamos los niveles de anticuerpos IgG frente a la proteína *Spike* del SARS-CoV-2 en la leche y el suero de mujeres lactantes vacunadas, para investigar la posible correlación entre ambos factores.

## Materiales y métodos

El presente estudio fue aprobado por el Comité Ético Institucional. Se realizó un estudio transversal en el que se incluyó a 180 participantes para evaluar los posibles cambios en los niveles de anticuerpos IgG frente a la proteína *Spike* del SARS-CoV-2 en mujeres en periodo de lactancia en Pakistán, entre el 1 de junio de 2021 y el 31 de agosto del mismo año. De esta muestra, 21 participantes completaron el estudio. Todas las participantes recibieron dos dosis de la vacuna de Sino pharm o Sinovac, con una diferencia de 21 días. Se tomaron muestras pareadas de leche y suero materno previamente a la vacunación (Momento 1) y 21 días después de la segunda dosis de la vacuna (dosis de refuerzo) (Momento 2). Todas las participantes firmaron un consentimiento informado previamente a la toma de las muestras. El estado de vacunación se validó mediante un certificado de vacunación. Se excluyó a las participantes con antecedentes de infección previa por COVID-19.

### Obtención de las muestras de leche y suero

Se emplearon los protocolos convencionales para la obtención de las muestras de leche y suero. Por otro lado, el análisis de anticuerpos frente a SARS COV2 se realizó mediante inmunoensayo de electroquimioluminiscencia (ECLIA) (Suiza), que se fundamenta en el principio del ensayo *sandwich* de doble antígeno (Elecsys Anti-SARS-CoV-2 S; Roche Switzerland), para cuantificar los niveles de anticuerpos frente a la proteína *Spike* del SARS-CoV-2. Las muestras se analizaron en una plataforma Cobas e801.

Las muestras de leche materna se extrajeron en el propio domicilio de las participantes usando un extractor de leche, y se enviaron al laboratorio para su posterior conservación, procesamiento y centrifugado.

Tras eliminar la capa de grasa, se analizó el sobrenadante. Las muestras de sangre se introdujeron en tubos de citrato ácido dextrosa, citrato de sodio, EDTA de potasio, y EDTA de tri potasio o de heparina de litio. Las muestras se incuban con antígenos RBD biotinilados y rutenilados, tras lo cual se añaden micropartículas recubiertas de estreptavidina, que se transfirieren a la célula a medir, donde se captan magnéticamente las micropartículas hacia el exterior del electrodo. Los fabricantes recomiendan un punto de corte de 0,8 U/mL para los anticuerpos en suero, por encima del cual el test se considera positivo, y viceversa.

Se observó linealidad en el intervalo de medición diagnóstica entre 0,4 y 250 U/mL. Dado que ELISA aún no se ha aprobado para muestras de leche materna, no establecimos ningún punto de corte. La leche materna es un tipo de muestra más heterogéneo que el suero, por lo que, para evitar interferencias, se restó de cada resultado la concentración media de anticuerpos en la leche de mujeres no vacunadas (grupo control).

Se aplicó estadística descriptiva para las variables demográficas. Se realizó un análisis de correlación entre las concentraciones de anticuerpos en suero y leche materna y el estado de inmunización calculando el coeficiente de correlación de Pearson.

## Resultados

La edad media de las participantes fue de 31 años (intervalo 24–38), mientras que los lactantes tenían una media de edad de 17 meses (intervalo 10–24 meses) (Tabla 1). Los análisis revelaron que la totalidad de las participantes presentaban anticuerpos frente a la proteína *Spike* del SARS-CoV-2.

**Tabla 1:** Características demográficas de las madres y los lactantes

Variables	
Participantes, n	21
**Media de edad materna, años**	31
Intervalo, años	24–38
**Media de edad de los lactantes. meses**	17
Intervalo, meses	10–24

Los niveles de anticuerpos se expresan en U/mL. El 100% de las participantes presentaban títulos de anticuerpos positivos (>128 U/mL) en la leche materna tras la vacunación. El 85% de las participantes presentaron anticuerpos en suero >0,8 U/mL 21 días después de haber recibido la segunda dosis de refuerzo (Tabla 2). Se observó una respuesta inmunogénica significativa en las muestras de leche materna tras la inmunización, comparadas con las muestras previas, con diferencias estadísticamente significativas (128 vs. 0,2) (p=0,001). También se halló una diferencia estadísticamente significativa en relación a la concentración de anticuerpos entre el suero materno antes y después de la inmunización (valor p 0,001) (Tabla 3). La concentración de IgG en suero y leche materna está directamente relacionada (valor p 0,001) (Tabla 4).

**Tabla 2:** Concentraciones de anticuerpos en el suero y la leche materna antes y después de la vacunación.

Concentración de IgG, U/mL	Suero	Pre-vacunación (momento 1)	0,1 U/mL
Concentración de IgG, U/mL	Suero	Pre-vacunación (momento 2)	54 U/mL
Concentración de IgG, U/mL	Leche materna	Pre-vacunación (momento 1)	0,2 U/mL
Concentración de IgG, U/mL	Leche materna	Pre-vacunación (momento 2)	128 U/mL

**Tabla 3:** Correlación entre la vacunación materna y los niveles de anticuerpos en el suero y leche materna.

Variables	Muestra	rs	Valor p
Pre-vacunación	Leche materna	0,8	0,236
Post-vacunación	Leche materna	0,141	0,001
Pre-vacunación	Suero	−0,11	0,64
Post-vacunación	Suero	0,48	0,001

**Tabla 4:** Correlación entre la concentración de IgG en suero y la leche materna.

Variables	rs	Valor p
Correlación entre la concentración de IgG en suero y la leche materna	0,84	0,001

## Discusión

Se debe determinar la respuesta inmune de los anticuerpos frente a la proteína *Spike* del SARS-CoV-2 en las mujeres en periodo de lactancia, para investigar la posible protección que confiere la vacunación materna frente a la COVID 19. Realizamos un análisis serológico a 21 participantes para determinar cuantitativamente los anticuerpos IgG frente a la proteína *Spike* del SARS-CoV-2 en el suero y la leche materna de una población paquistaní.

En el momento del estudio, la OMS había aprobado dos vacunas contra el SARS-CoV-2 para mujeres en periodo de lactancia [4]. Se observaron niveles de anticuerpos contra el SARS-CoV-2 significativamente mayores en la leche de todas las participantes, lo que confirma los resultados de otros estudios [15]. Así mismo, también hallamos un aumento significativo en los niveles de anticuerpos IgG frente al SARS-CoV-2 tras la administración de la segunda dosis de refuerzo, lo que va en consonancia con los resultados obtenidos en un estudio realizado en 84 mujeres en Israel [16]. Estos prometedores resultados refuerzan la teoría de que la inmunización de las mujeres en periodo de lactancia puede tener un efecto protector en sus hijos o hijas lactantes, tal como demuestra un estudio realizado en Moscú en 17 mujeres lactantes [17]. En otro estudio realizado en madres en periodo de lactancia en España, se observaron niveles elevados de anticuerpos tanto en el suero como en la leche de las madres en periodo de lactancia [18]. Del mismo modo, otro estudio realizado en Polonia en 32 profesionales sanitarias en periodo de lactancia reveló una correlación significativa entre los anticuerpos IgG frente a SARS COV2 en las muestras de leche y suero materno [19].

Los resultados del presente estudio son relevantes, no únicamente para planificar estratégicamente la inmunización a nivel organizacional y nacional, sino que también revelan la necesidad de determinar los niveles de anticuerpos frente a la proteína *S* *pike* del SARS-CoV-2, en las madres en periodo de lactancia a nivel global. Este estudio se basa en una pequeña muestra.

## Conclusiones

A continuación, se describen las implicaciones de los hallazgos del presente estudio:

1. Todas las mujeres en periodo de lactancia desarrollan inmunidad tras la vacunación, elevándose sustancialmente los niveles de anticuerpos tras recibir la dosis de refuerzo.
2. Se observó una fuerte correlación positiva entre los anticuerpos IgG frente a SARS COV2 en las muestras de suero y leche materna.
3. Aunque son necesarios más estudios, los hallazgos de este estudio podrían tener un impacto significativo en la inmunización de este grupo de población.
4. Así mismo, estos hallazgos también señalan una estrategia de inmunización de los lactantes frente a la COVID 19.
